# High plasma complement C4 levels as a novel predictor of clinical outcome in intracerebral hemorrhage

**DOI:** 10.3389/fnagi.2023.1103278

**Published:** 2023-02-20

**Authors:** Moxin Wu, Kai Chen, Min Jiang, Fusheng Xie, Xianming Cao, Liang Chen, Zhiying Chen, Xiaoping Yin

**Affiliations:** ^1^Department of Medical Laboratory, Affiliated Hospital of Jiujiang University, Jiujiang, China; ^2^Jiujiang Clinical Precision Medicine Research Center, Jiujiang, China; ^3^Department of Dermatology, Wuhan No. 1 Hospital, Tongji Medical College, Huazhong University of Science and Technology, Wuhan, China; ^4^Department of Neurology, Affiliated Hospital of Jiujiang University, Jiujiang, China; ^5^Department of Radiology, Affiliated Hospital of Jiujiang University, Jiujiang, China

**Keywords:** brain edema, complement component 4, intracerebral hemorrhage, predictor, second brain injury

## Abstract

**Objective:**

The complement cascade is activated and contributes to the brain injury after intracerebral hemorrhage (ICH). Complement component 4 (C4), an important component of complement cascade, has been associated with severity of neurological impairment that occurs during ICH. However, the correlation of plasma complement C4 levels with hemorrhagic severity and clinical outcome in ICH patients has not been reported.

**Materials and methods:**

This study is a monocentric, real-world, cohort study. In this study, we measured the plasma complement C4 levels of 83 ICH patients and 78 healthy controls. The hematoma volume, the National Institutes of Health Stroke Scale (NIHSS) score, the Glasgow Coma Scale (GCS) score, and the permeability surface (PS) were used to assess and quantify neurological deficit following ICH. Logistic regression analysis was configured to determine the independent relation of plasma complement C4 levels to hemorrhagic severity and clinical outcomes. The contribution of complement C4 to secondary brain injury (SBI) was assessed by changes in plasma C4 levels between admission and at day 7 after ICH.

**Results:**

There was a significant elevation of plasma complement C4 levels in ICH patients than in healthy controls (40.48 ± 1.07 vs. 35.25 ± 0.60, *p* < 0.0001), and the plasma complement C4 levels were closely related to the hemorrhagic severity. Moreover, plasma complement C4 levels of patients were positively correlated with the hematoma volume (*r* = 0.501, *p* < 0.001), NIHSS score (*r* = 0.362, *p* < 0.001), the GCS score (*r* = −0.490, *p* < 0.001), and PS (*r* = 0.683, *p* = 0.045) following ICH. Logistic regression analysis also confirmed that patients with high plasma complement C4 levels show a poor clinical outcome after ICH (*p* < 0.001). Meanwhile, the elevated plasma levels at day 7 after ICH indicated the correlation of complement C4 with SBI (*p* < 0.01).

**Conclusion:**

Plasma complement C4 levels are significantly elevated in ICH patients and positively correlated with the illness severity. Thus, these findings highlight the importance of complement C4 in brain injury after ICH and provide a novel predictor of clinical outcome for this disease.

## Introduction

Intracerebral hemorrhage (ICH) is a common cerebrovascular disease and characterized by the neurological impairment and brain injury with high mortality and disability rates in patients (Hostettler et al., 2019). Complement system, also known as complement cascade, is an essential part of innate immune system and plays an important role in brain injury (Dalakas et al., 2020; Iadecola et al., 2020). Several researches have reported that the complement-mediated brain injury in many neurodegenerative diseases, such as Alzheimer’s disease (AD), traumatic brain injury (TBI), and cerebral ischemia and ICH (Shi et al., 2017; Alawieh et al., 2018b; Schartz and Tenner, 2020). During the early phase after ICH, the complement cascade can be activated through classical, lectin, or alternative pathway and contributes to inflammation, neuronal cell death, and brain edema, thus promoting secondary brain edema and injury after ICH (Dalakas et al., 2020; Schartz and Tenner, 2020). Moreover, a number of previous studies have demonstrated the complement cascade activation and membrane attack complex (MAC) formation after ICH (Yao et al., 2017; Yuan et al., 2017; Wang et al., 2019). However, existing data are still limited and further investigation is required to explore the potential utility of complement components as predictor of clinical outcome for ICH patients.

Complement component 4 (C4) is an essential part of the cascade, which leads to component C3 activation in the classical/lectin pathway, involving the cleavage of C4 into C4a and C4b. Some clinical studies have shown the increased complement C4 levels in the plasma of patients with schizophrenia and major depressive disorder (MDD) (Wei et al., 2018; Comer et al., 2020), and high plasma complement C4 can also be used as a predictor of unfavorable outcomes in diabetic stroke (Zhang et al., 2021). Moreover, a recent transcriptomic study revealed the continued upregulation of complement C4 expression in humans with TBI (Toutonji et al., 2021). Clearly, deficiency in complement C4 reduced brain tissue damage and improved post-injury motor deficits after TBI in mice (You et al., 2007). Meanwhile, elevated serum component C3 levels increased risks of adverse clinical outcomes after ischemic stroke, and C3 depletion attenuated brain edema and neutrophil infiltration around the clot after ICH (Yang et al., 2006, 2021). Taken together, these findings highlight the importance of complement C4 in brain injury and implicate the potential role of complement C4 in ICH. However, no studies have examined the complement C4 levels in peripheral blood of ICH patients.

In this study, we measured the plasma complement C4 levels in patients with ICH and evaluated whether plasma complement C4 levels are associated with illness severity following ICH, and further determined the potential utility of plasma complement C4 as a potential predictor of clinical outcome of human ICH.

## Materials and methods

### Study design

This real-world, retrospective cohort study was conducted of patients with first-ever acute spontaneous ICH admitted to the Affiliated Hospital of Jiujiang University (Jiujiang, China) diagnosed *via* head computed tomography (CT) scans from January 2021 to May 2022. All patients were hospitalized within 24 h after stroke, and their hematomas were received non-operative treatment. The exclusion criteria were: (1) less than 18 years old; (2) surgical treatment indicated; (3) ICH resulting from TBI, hemorrhagic transformation of cerebral infarction, intracranial aneurysm, intracranial tumors, arteriovenous malformation, venous sinus thrombosis, or moyamoya disease; (4) pre-ICH modified Rankin scale (mRS) ≥ 2; and (5) other specific conditions, such as severe infections within recent a month, known malignancies, and autoimmune diseases. At the same time, a group of healthy volunteers matched in age and gender were selected as controls. The control group underwent routine laboratory tests, and had no history of surgery and other diseases such as hypertension, diabetes mellitus, malignancies, and stroke. This study was approved by the Institutional Review Boards at Affiliated Hospital of Jiujiang University (Grant No. IRB2022-JJU-032-21). The written informed consent for participating was signed by patients or their immediate family members and controls themselves.

### Data collection

Peripheral venous blood samples of ICH patients were collected after at least 8 h within 24 h of patients’ hospital admission and 7 days after ICH. Those of healthy controls were collected at enrollment in our hospital from January 2022 to May 2022. Relevant information such as age, gender, vascular risk factors (hypertension and diabetes mellitus), cigarette smoking, alcohol consumption, leucocyte count, blood glucose, and potassium level were collected. The Siemens Leonardo V software for semiautomatic CT volumetry has been used for assessment of hematoma and perihemorrhagic edema volumes (Kollmar et al., 2012). CT perfusion images were transferred to a workstation (Philips Healthcare) to generate perfusion parameter maps of the cerebral blood flow, cerebral blood volume, time to peak, mean transit time and permeability surface (PS).

### Plasma C4 levels detection

For measurement of plasma complement C4 levels, blood samples were centrifuged at 3000 g for 10 min, and afterward, plasma samples were separated at the clinical laboratories and immediately frozen at –80°C for subsequent measurement. Plasma complement C4 concentrations were quantitatively measured following the manufacturer’s package inserts for Beckman Coulter reagents on the Beckman Coulter AU5800 clinical chemistry analyzer (Beckman Coulter Inc., Brea, CA, United States). The measurement was completed by the same technician, who was inaccessible the clinical data.

### Clinical outcome assessment

The disease severity was assessed utilizing the National Institutes of Health Stroke Scale (NIHSS) score and the Glasgow Coma Scale (GCS) score by trained neurologists at admission (Currò et al., 2022; Wan et al., 2022).

### Statistical analysis

Statistical analysis was carried out using GraphPad Prism version 9.0 (GraphPad Software Inc., La Jolla, CA, United States). Kolmogorov–Smirnov test or Shapiro–Wilk test was conducted to determine normal distribution of quantitative data. Normally distributed data were reported as means ± standard deviations and non-normally distributed data were summarized as medians with upper and lower quartiles. The categorical variables were presented as number of cases (percentages) and the comparison between two groups was performed by the unpaired *t*-tests, paired *t*-test, or Mann–Whitney *U* rank-sum test as appropriate. Data among multiple groups were compared using the Kruskal–Wallis *H*-test. The categorical variables were presented as number of cases (percentages) and the comparison between two groups was performed by the Fisher’s exact test or *χ*^2^ test. The median value of the hematoma volume, NIHSS score, and GCS score was defined as the cutoff value, respectively. The Spearman’s correlation coefficient was carried to analyze bivariate correlations, the association between plasma complement C4 level and clinical outcomes was assessed by multivariate logistic regression, and *p*<0.05 was considered to be statistically significant.

## Results

### Patient selection and characteristics

**Figure 1:**
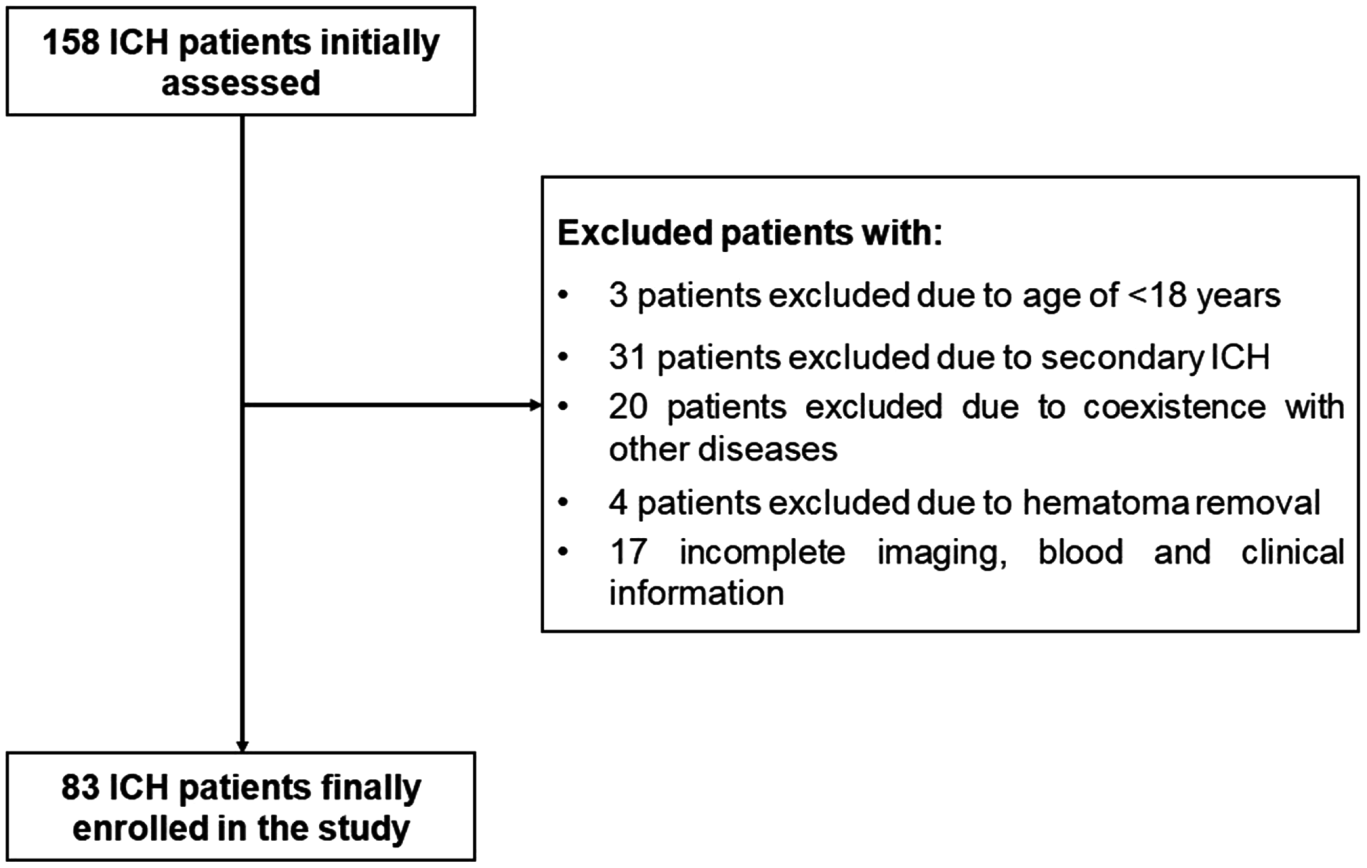
The flowchart for screening eligible patients with acute spontaneous ICH. Initially, 158 ICH patients were assessed; thereafter, we excluded 75 patients; and ultimately, 83 ICH patients were enrolled. ICH indicates intracerebral hemorrhage.

During the study, a total of 158 first-ever acute spontaneous ICH patients hospitalized within 24 h after onset of symptom were initially included in the study. Afterward, 75 patients were excluded according to the exclusion criteria, as shown in Figure 1. Finally, 83 ICH patients (56 males and 27 females) were enrolled in this study. In addition, 78 healthy controls (51 males and 27 females) were recruited. The average age of the ICH patients and healthy controls was 63.73 ± 13.52 and 64.13 ± 11.86 years, respectively. There was no statistically significant difference of gender (*p* = 0.779), age (*p* = 0.834), current smokers (*p* = 0.687), and alcohol drinkers (*p* = 0.698) between the ICH patients and healthy controls. Expectedly, ICH patients had higher percentages of hypertension and diabetes mellitus compared to healthy controls (*p* < 0.001), as well as were more likely to have significantly elevated plasma glucose level (9.21 ± 2.94 vs. 5.44 ± 0.92, *p* < 0.001), blood leucocyte count (9.64 ± 4.47 vs. 5.93 ± 1.38, *p* < 0.001), and a lower plasma potassium level (3.79 ± 0.54 vs. 4.00 ± 0.30, *p* < 0.01). Collectively, the demographics and clinical characteristics of the patients are provided in Table 1.

**Table 1:** Demographic data and vascular risk factors of the intracerebral hemorrhage (ICH) patients and control patients in this study.

**Characteristic**	**Control (*n* = 78)**	**ICH (*n* = 83)**	***p*-value**
Male, n (%)	51 (65.38%)	56 (67.47%)	0.779
Age, years (mean ± SD)	64.13 ± 11.86	63.73 ± 13.52	0.834
Hypertension, *n* (%)	0	67 (80.72%)	< 0.001*
Diabetes mellitus, *n* (%)	0	12 (14.46%)	< 0.001*
Current smoking, *n* (%)	19 (24.36%)	18 (21.69%)	0.687
Alcohol consumption, *n* (%)	14 (17.95%)	13 (15.66%)	0.698
Plasma glucose level (mmol/L)	5.44 ± 0.92	9.21 ± 2.94	< 0.001*
Plasma potassium level (mmol/L)	4.00 ± 0.30	3.79 ± 0.54	< 0.01*
Blood leucocyte count (×10^9^/L)	5.93 ± 1.38	9.64 ± 4.47	< 0.001*

Age was reported as the mean ± standard deviation and qualitative data were presented as counts (proportions). Intergroup comparisons of various variables were conducted using the *χ*^2^ test for qualitative data, and Mann–Whitney *U*-test for quantitative data. The asterisk indicates statistical significance (*p < 0.05).

### Elevated plasma C4 levels and Its correlation with clinical outcomes

**Figure 2:**
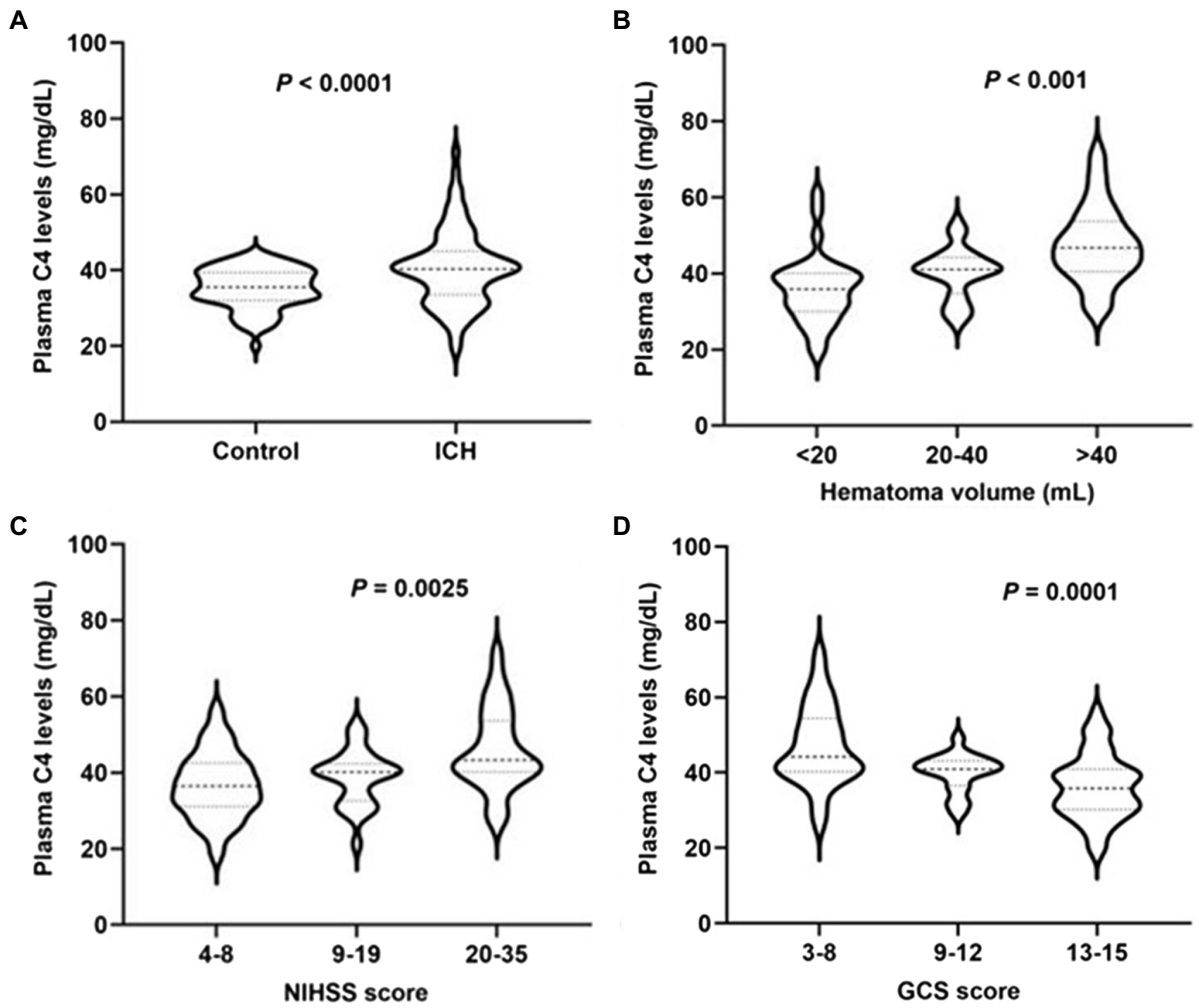
High plasma complement C4 levels in ICH patients and are associated with hemorrhagic severity. Plasma C4 levels were reported as median (upper-lower quartiles). **(A)** Plasma complement C4 level in ICH and healthy controls. Association of plasma C4 levels to **(B)** hematoma volume, **(C)** NIHSS score, and **(D)** GCS score after acute ICH patients. ICH, intracerebral hemorrhage; NIHSS, National Institutes of Health Stroke Scale; GSC, Glasgow Coma Scale.

Plasma complement C4 levels in patients with ICH were significantly higher than those in healthy controls (40.48 ± 1.08 vs. 35.25 ± 0.60, *p* < 0.0001) (Figure 2A). In order to discern the relationship between plasma complement C4 levels and hemorrhagic severity following ICH, we analyzed the hematoma volume, NIHSS score, and the GCS score of ICH patients. Interestingly, we found that the plasma complement C4 levels were markedly higher in patients with hematoma volume of 20–40 ml than in those with hematoma volume less than 20 ml, as well as in patients with hematoma volume more than 40 ml than in those with hematoma volume of 20–40 ml (*p* < 0.001) (Figure 2B). Similarly, compared to patients with lower NIHSS score and GCS score, those presenting with higher NIHSS score and GCS score had significantly elevated plasma complement C4 levels (NIHSS score, *p* = 0.0025; GCS score, *p* = 0.0001) (Figures 2C,D). Taken together, our results demonstrated that plasma complement C4 levels are significantly elevated in ICH patients and closely related to the hemorrhagic severity.

**Figure 3:**
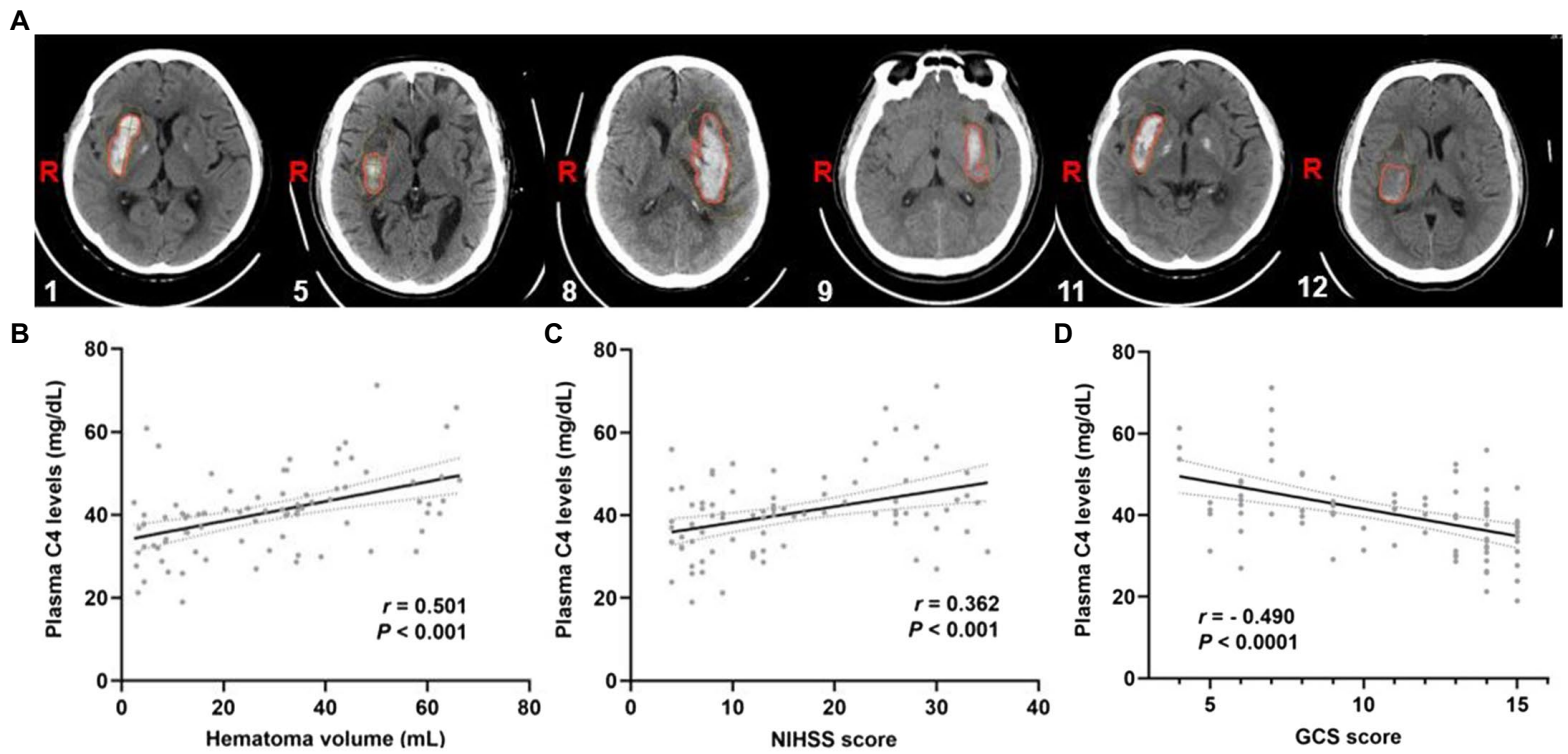
Plasma complement C4 levels are positively correlated with the clinical outcomes of the ICH patients. **(A)** CT images of the ICH patients used for analysis, the corresponding serial number of the patients are listed in the lower left corner of each image. Correlation analysis between the plasma C4 level and **(B)** hematoma volume, **(C)** the NIHSS score, and **(D)** GCS score of the ICH patients (*n* = 83). Correlations were done using the Spearman’s correlation coefficient in ICH, plasma C4 levels were closely correlated with hematoma volume, NIHSS score, and GCS score. ICH, intracerebral hemorrhage; NIHSS, National Institutes of Health Stroke Scale; GSC, Glasgow Coma Scale.

To further explore the importance of elevated plasma complement C4 levels in hemorrhagic severity, Spearman’s correlation coefficient was used to identify correlations between plasma complement C4 levels and hematoma volume, NIHSS score, and GCS score. Figure 3A shows the representative computed tomography (CT) imaging diagnoses of the patients used for analysis. The analysis of plasma complement C4 levels and clinical outcomes revealed that complement C4 levels are positively correlated with the hematoma volume (r = 0.501, *p* < 0.001), NIHSS score (r = 0.362, *p* < 0.001), and the GCS score (r = −0.490, *p* < 0.001) following ICH (Figures 3B–D). Moreover, high plasma complement C4 levels showed more hematoma volume (OR, 1.11; 95% CI, 1.05–1.19; *p* < 0.0001), more serious NIHSS score (OR, 1.12; 95% CI, 1.06–1.20; *p* < 0.0001), and GCS score (OR, 0.73; 95% CI, 0.62–0.84; *p* = 0.0001) in the multiple logistic regression analysis (Table 2). These findings support the conclusion that high plasma complement C4 levels are positively correlated with the neurological deficit severity and clinical outcomes in ICH patients.

**Table 2:** Multivariate logistic regression analysis for risk factors of poor clinical outcomes after ICH.

**Logistic analysis (*p*-value)**	**Hematoma volume**	**NIHSS score**	**GCS score**
Age	0.231	0.1789	0.5357
Gender	0.2071	0.2546	0.0323*
Hypertension	0.1401	0.788	0.4281
Diabetes mellitus	0.6852	0.6453	0.7258
Current smoking	0.6752	0.5265	0.9673
Alcohol consumption	0.2455	0.2237	0.1376
Plasma glucose level	0.5178	0.3841	0.5477
Plasma potassium level	0.2764	0.5783	0.8949
Blood leucocyte count	0.1567	0.0049*	0. 0404*
Plasma C4 level	< 0.0001*	< 0.0001*	0.0003*

C4, component 4. The asterisk indicates statistical significance (*p < 0.05).

### The association between elevated plasma C4 levels and SBI

**Figure 4:**
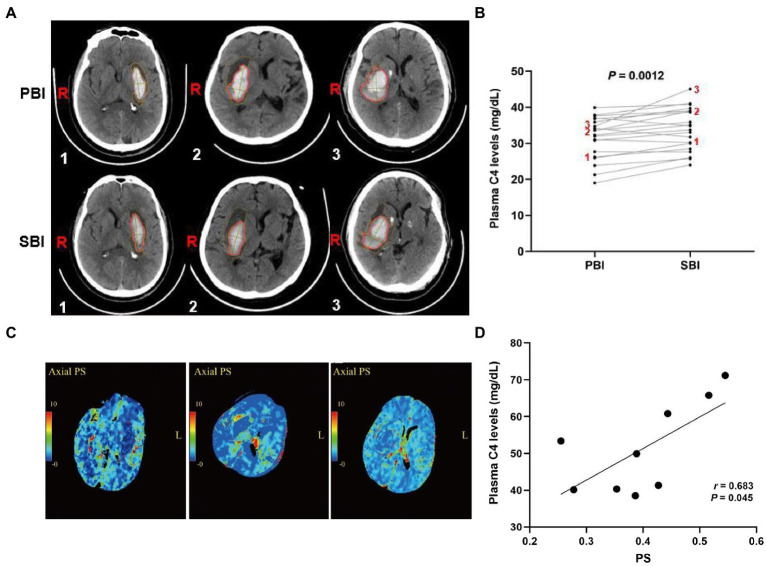
Elevated plasma complement C4 levels contribute to secondary brain injury after ICH. **(A)** CT images of the ICH patients in PBI and SBI and **(B)** plasma complement C4 levels in PBI and SBI using paired *t*-test (n = 20), **(C)** Representation of PS values at 24 h in patients with severe SBI, and **(D)** correlation between PS value and C4 levels at 24 h in patients with severe SBI. ICH, intracerebral hemorrhage; PBI, primary brain injury; SBI, secondary brain injury, PS, permeability surface.

Activated complement cascade promotes erythrolysis and secondary perihemorrhagic edema (Wang et al., 2019; Holste et al., 2021), we then assayed the relationship between plasma complement C4 levels and SBI after ICH. Representative CT images clearly showed the reduced hematoma volume and expanded edema volume of the patients in SBI (Figure 4A). We then tested plasma complement C4 levels of the patients. Surprisingly, compared with primary brain injury (PBI), we found that almost every patient displayed a significant increase in SBI (Figure 4B), indicating a positive correlation between elevated plasma complement C4 levels and SBI after ICH. We analyzed the PS values and complement C4 of these SBI patients on admission and found that C4 was also at its peak (Figures 4C,D). We estimated that C4 may aggravate SBI by affecting the blood-brain barrier (BBB).

## Discussion

Growing evidence indicates the contribution of complement-mediated brain injury in the neurological deficit after ICH (Alawieh et al., 2018a; Ma et al., 2019; Petrisko et al., 2021). Among all complement components, the classical complement initiator C1q and the central complement C3 are the most widely and deeply investigated cascades (Wang et al., 2021, 2022; Zheng et al., 2022). However, as an essential cascade in the activation of classical and lectin pathways (You et al., 2007), the role of complement C4 in ICH remains elusive. This is the first study that thoroughly examined complement C4 levels in the plasma of patients with ICH. Our results found that ICH patients have significantly higher plasma complement C4 levels than healthy controls, but the association between elevated plasma complement C4 levels and neurologic function is still unknown. In this study, we found that plasma complement C4 levels are closely related to the hematoma volume of patients, and those patients with higher plasma complement C4 levels also have higher NIHSS score and GCS score, indicating the poor neurological scores in these patients. In line with our data, high serum complement C4 levels as a unique predictor were associated with clinical outcomes in diabetic stroke patients (Zhang et al., 2021). Moreover, a previous clinical study revealed that serum complement C4 levels were elevated and associated with GCS score in patients with anti-N-methyl-d-aspartate receptor (NMDAR) encephalitis (Shu et al., 2018). Taken together, our data not only provide direct evidence for the elevated plasma complement C4 levels in ICH patients but also suggest the detrimental effect of excessive activation of C4 to neurological function.

We also performed logistic regression analysis and found that the plasma C4 levels were positively correlated with the hemorrhagic severity and clinical outcomes of the patients. These data indicated the contribution of high plasma C4 levels to the brain injury after ICH, but the link between elevated plasma C4 levels and ICH pathology remains unclear. As reported, complement activation and opsonization at hippocampal synapses direct ongoing microglia-dependent phagocytosis of synapses and lead to a loss of synaptic density after ischemic stroke. Inhibition of complement activation or phagocytosis by microglia recuses synaptic loss, attenuates brain injury, and improves neurobehavioral outcomes after hemorrhagic stroke (Alawieh et al., 2020; Shi et al., 2021). More importantly, C4 is critical to synaptic pruning, and variation of C4 induces an excessive neuronal complement deposition and contributes to increased microglial synapse uptake in co-cultured human iPSC-derived neurons and microglial cells (Sellgren et al., 2019). Moreover, based on the genome-wide association study of largest population, Sekar and his colleagues found that C4 is expressed at higher levels during the synaptic refinement period of dorsolateral geniculate nucleus, and C4-deficient mice show defects in synaptic elimination in the retinogeniculate system (Sekar et al., 2016). Similarly, two animal model studies revealed that enhanced expression of C4 promotes excessive synaptic loss and microglia synaptic engulfment in mice (Comer et al., 2020; Yilmaz et al., 2021). Thus, it is likely that C4-mediated brain injury *via* regulating the microglia-dependent synaptic engulfment in ICH, but the exact role of C4-mediated synaptic phagocytosis in the pathogenesis of ICH deserves further investigation.

Complement cascades not only acts an essential role in the pathogenesis of neurological deficit in the acute primary of ICH, but also appears to have critical implication for SBI in the chronic phase (Chen et al., 2013; Alawieh et al., 2018a). In this study, we observed a significant increased plasma C4 levels in the chronic secondary phase of ICH, suggesting an association between elevated plasma C4 levels and SBI after ICH. We retrospectively collected the CT perfusion images of patients with severe SBI within 24 h after admission, and the permeability surface area was extracted. We found that these patients already had elevated PS values at an early stage. Analysis of C4 on the first day showed that C4 was also at its peak. We estimated that C4 may aggravate SBI by affecting the BBB. It has been reported that neuroinflammation is an important component of brain injury and the complement cascade has been recognized as one contributor to neuroinflammation and secondary insult through promoting neuronal death, brain edema, and (BBB) breakdown (Bellander et al., 2011; Brennan et al., 2012; Schartz and Tenner, 2020). Two previous investigations showed that complement system is implicated in triggering neuroinflammation and tackling neuroinflammation *via* complement inhibition as a useful therapy for secondary injury after TBI (Alawieh et al., 2018b; Van Erp et al., 2022). Similarly, two previous studies revealed that elevated complement cascades levels are associated with acute neuroinflammation and blocking complement activation reduces the extent of secondary brain damage (Neher et al., 2014; Holden et al., 2021). Simultaneously, another study reported that increased expression of complement C4 contributes to the local inflammatory state in AD patients (Zorzetto et al., 2017). We have hypothesized that plasma complement C4 is activated quickly after ICH and may contribute to brain edema and secondary brain injury in multiple ways including inflammatory cytokines and activation of microglia, iron overload, and synaptic pruning. In future, we need to disrupt complement C4 levels in animal models of ICH using pharmacological blockade or genetic deletion to further elucidate its effect on secondary brain injury after ICH. Collectively, these evidences strongly implicate the pivotal role of elevated plasma C4 levels in the neuroinflammation after ICH, and inhibition of overshooting activation of C4 might be a potential strategy to reduce secondary brain injury for this disease.

In conclusion, this study for the first time provided the significantly higher plasma C4 levels in ICH patients; and plasma C4 levels are positively correlated with the clinical outcomes of patients and also increased at SBI following ICH, suggesting that C4 may be involved in SBI after ICH and plasma C4 levels could be a promising novel prognostic biomarker for this disease.

## Data availability statement

The raw data supporting the conclusions of this article will be made available by the authors, without undue reservation.

## Ethics statement

This study was approved by the Institutional Review Boards at Affiliated Hospital of Jiujiang University (Grant No. IRB2022-JJU-032-21). Written informed consent to participate in this study was provided by the participants’ legal guardian/next of kin.

## Author contributions

XY and ZC were responsible to conceive and design the study. MW and KC developed the method, performed data visualization, statistical analysis, and prepared the manuscript. MJ and FX acquired, collected, and extracted the data included in this analysis. XC, MW, and LC analyzed the data. KC, MW, and ZC helped with manuscript preparation and data review. The final version of the manuscript was approved by all authors. All authors contributed to the article and approved the submitted version.

## Funding

This study was supported partially by the National Natural Science Foundation of China (82260209 and 81960221 to XY, 82203926 to KC); the National Science and Technology Fundamental Resource Investigation Program of China (2018FY100903 to XY); Science and Technology Project Founded by the Education Department of Jiangxi Province (GJJ201834 to MW); Jiangxi Provincial Health Commission Science and Technology Plan project (202212021 to MW, 202311506 to ZC); Hubei Provincial Natural Science Foundation of China (2022CFB955 to KC); and Jiangxi Provincial Administration of Traditional Chinese Medicine Science and Technology Plan Project (2022A322 to ZC).

## Conflict of interest

The authors declare that they have no known competing financial interests or personal relationships that could have appeared to influence the work reported in this paper.

## Publisher’s note

All claims expressed in this article are solely those of the authors and do not necessarily represent those of their affiliated organizations, or those of the publisher, the editors and the reviewers. Any product that may be evaluated in this article, or claim that may be made by its manufacturer, is not guaranteed or endorsed by the publisher.
